# Non-viral gene therapy using RNA interference with PDGFR-α mediated epithelial-mesenchymal transformation for proliferative vitreoretinopathy

**DOI:** 10.1016/j.mtbio.2023.100632

**Published:** 2023-04-11

**Authors:** Jiahao Wang, Peiyi Zhao, Zhirong Chen, Hui Wang, Yajia Wang, Quankui Lin

**Affiliations:** Department of Biomaterials, School of Ophthalmology & Optometry, Eye Hospital, Wenzhou Medical University, Wenzhou, 325027, PR China

**Keywords:** Non-viral vector, PEI-g-PEG, Proliferative vitreoretinopathy, Epithelial-mesenchymal transformation, PDGFR-α

## Abstract

Fibrotic eye diseases, a series of severe oculopathy, that will destroy normal ocular refractive media and imaging structures. It is characterized by the transformation of the epithelial cells into mesenchyme cells. Proliferative vitreoretinopathy (PVR) is one of these representative diseases. In this investigation, polyethylene glycol grafted branched Polyethyleneimine (PEI-*g*-PEG) was used as a non-viral gene vector in gene therapy of PVR to achieve anti-fibroblastic effects in vitro and in vivo by interfering with platelet-derived growth factor alpha receptor (PDGFR-α) in the epithelial-mesenchymal transition (EMT) of retinal pigment epithelium (RPE) cells. The plasmid was wrapped by electrostatic conjugation. Physical characterization of the complexes indicated that the gene complexes were successfully prepared. In vitro, cellular experiments showed excellent biocompatibility of PEI-g-PEG, efficient cellular uptake of the gene complexes, and successful expression of the corresponding fragments. Through gene silencing technique, PEI-g-PEG/PDGFR-α shRNA successfully inhibited the process of EMT in vitro. Furthermore, in vivo animal experiments suggested that this method could effectively inhibit the progression of fibroproliferative membranes of PVR. Herein, a feasible and promising clinical idea was provided for developing non-viral gene vectors and preventing fibroblastic eye diseases by RNA interference (RNAi) technology.

## Graphical abstract

Hyperbranched pegylated polycationic PEI-*g*-PEG is designed to prevent PVR by encapsulating nucleic acid drugs through electrostatic association and delivering Short hairpin RNA plasmids to epithelial mesenchymal transition cells. PEI-g-PEG as a non-viral gene vector significantly increases biosafety and cellular uptake, and the accompanying delivered PDGFR-α shRNA plasmid can prevent fibroproliferative membrane production in PVR. This process interferes with protein expression through a gene silencing process targeted to block growth factor receptor-mediated epithelial mesenchymal transition.

**Figure:**
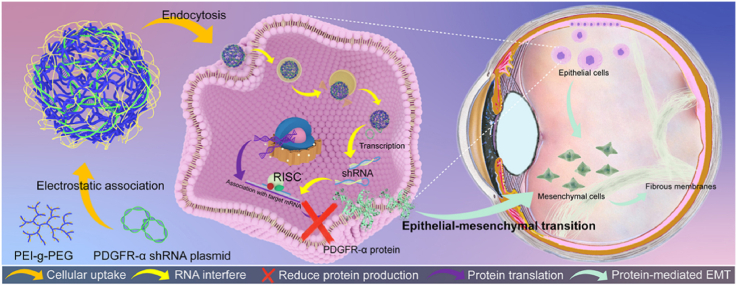

## 1 Introduction

A common feature of fibrotic eye diseases is the transdifferentiation of cells into mesenchymal cells, termed EMT [1,2]. While EMT is essentially a bodily recovery process, wound healing can result in the dysfunctional, diseased fibrous tissue that causes varying degrees of damage to surrounding tissues and organs, depending on the location and extent of recovery [3,4]. For the eye, this process does more harm than good. It will destroy the transparency of the refractive medium, impair normal refractive power and result in visual impairment and blindness to millions of people worldwide [5]. Related diseases are numerous, including cornea scarring [6], pterygium [7], posterior capsule cataract (PCO) [8], PVR [9], secondary glaucoma and orbital fibrosis, etc [10]. Effective monitoring and targeting of EMT markers for related diseases are of great significance for the prevention of blindness [11,12]. Among them, PVR is one of the representative fibrous eye diseases that can lead to severe visual impairment and even blindness. It occurs in 5%–10% of all rhegmatogenous retinal detachments and 40%–60% of open-eye injuries [9,13,14]. It is characterized by the formation of contractible fibrous membranes behind the vitreous cavity and the anterior retina. The contraction and pulling of the membranes will cause retinal detachment [15]. Mature RPE cells are in a mitotic quiescent state under physiological conditions. Once the neural retina is damaged, RPE cells will begin to proliferate at the same time as transformation [13,16]. The epithelial-mesenchymal transition of the retinal pigment epithelium RPE cells has become the consensus in the pathophysiological process of disease formation [5]. Precise regulation of gene expression patterns is critical for the transformation and differentiation of epithelial cells into mesenchymal cells [17,18]. Therefore, anti-fibrotic therapies targeting cascade signal interference in EMT can be a promising therapeutic modality to reduce the incidence of fibrotic eye diseases developing into blindness.

This targeted interference with cascade signaling is different from traditional therapy such as the one using small molecules of drugs or antibodies. It is one of the nucleic acid (NA) therapies, which refers to the process of degrading messenger RNA and preventing translation into functional proteins. Briefly, it uses NA molecules as drugs that are fully complementary to the target mRNA with the help of an activated silencing complex (RISC), also known as RNAi-based therapeutics [[19], [20], [21]]. The implementation of RNAi is mediated by two types of molecules that include chemically synthesized double-stranded small interfering RNA (siRNA) and short hairpin transfer RNA (shRNA) [22,23]. Although siRNA and shRNA can be used to achieve similar functional effects, their molecular mechanisms of action, RNAi pathways, off-target effects, and applications are diverse [24,25]. The ease of preparation and short duration of siRNAs make them ideal for topical therapy. Also, the chemically synthesized siRNAs are easily modified by chemicals. However, extensive and complicated modifications to siRNAs can lead to higher production costs [23,26]. In addition, as its RNA is chemically synthesized, it is easier to degrade and has a shorter action time. So the therapeutic effect is unstable [27]. Unlike siRNAs, shRNA can be combined with artificial fluorescent tags, simple tags, and other eukaryotic taxonomic markers. Optimized shRNA constructs utilize endogenous processing machinery to achieve high-efficiency and long-lasting effects at low copy numbers and reduce off-target effects, especially when combined with miRNA scaffolds [22,25].

It is well known that nucleic acid molecules enter cells primarily through the mechanism of endocytosis [[28], [29], [30]], which requires the transport of the carrier to the cytoplasm to function. Viral vectors are the most potent NA vectors currently used to develop human therapeutics, including clinically approved retroviruses, lentiviruses, adenoviruses, and adeno-associated viruses (AAVs) [31,32]. However, the potential risk of immunogenicity and biosafety remains a great limitation for viral vectors [33,34]. Thus, the introduction of non-viral vectors has attracted great interest, containing liposomes, polymer nanoparticles, inorganic nanoparticles, dendrimers, DNA/RNA nanostructures, etc [19]. Those nanoparticle-based vehicles can not only improve gene complex stability, drug loading, and drug loading efficiency, but also prevent NA degradation and ensure controlled drug release [35]. Owing to improved therapeutic efficiency and reduced adverse effects, they are attractive for biomedical delivery and therapy [36,37]. Among them, polycationic nanocarriers are the most popular one. They have sufficient space to carry the nucleic acids with therapeutic effects due to the presence of functional groups such as amides and polyterminated amines [38,39]. For example, Polyethyleneimine (PEI) has been the commonly used cationic carrier of choice because of its good sponge proton effect in academia. The advantage of the “proton sponge effect” is that the gene complex accumulates in acidic vesicles, absorbs H^+^, and allows Cl^−^ and water to enter the interior to act as a buffer. The swelling and rupture of the lysosome make the gene escape into the cytoplasm [40]. Due to its high charge density and strong buffering capacity, PEI can help protect DNA from lysosomal cleavage [41,42]. However, PEI-based gene delivery systems are often highly cytotoxic [43,44]. A polyethylene glycol grafted branched PEI (PEI-g-PEG) was used instead of PEI to improve its performance and reduce toxicity [34]. This is mainly because the steric repulsion of the hydrophilic PEG segment can also shield excess positive charges and reduce the toxicity of PEI [38]. Additionally, the modification of the PEG segment can improve the stability of the complex and prolongs blood circulation [38]. Accordingly, the use of advanced nanotechnology to assist molecular biology in disease treatment is promising.

In recent years, the role of proliferating platelet-derived growth factor (PDGF) and its receptor tyrosine kinase in this EMT process has gained the attention of scholars [[45], [46], [47], [48]]. PDGF signaling has been implicated in fibrotic diseases involving progressive scarring and loss of tissue function due to mesenchymal cell proliferation and extracellular matrix deposition [49]. The activated proliferating PDGFR undergoes autophosphorylation and can interact with signaling proteins containing src homology 2 (SH2) domains, resulting in the activation of various signaling pathways, such as MAP kinase pathway, PI3-kinase-Akt, PLCγ, etc [[49], [50], [51], [52]]. In the vitreous cavity of PVR, including the secretion of PDGF by RPE cells themselves, non-PDGF components of the vitreous and vascular endothelial growth factor (VEGF), all of them work together to participate in the activation of PDGFR-α and thus determine the pathological evolution of PVR [49,53,54].

**Scheme 1:**
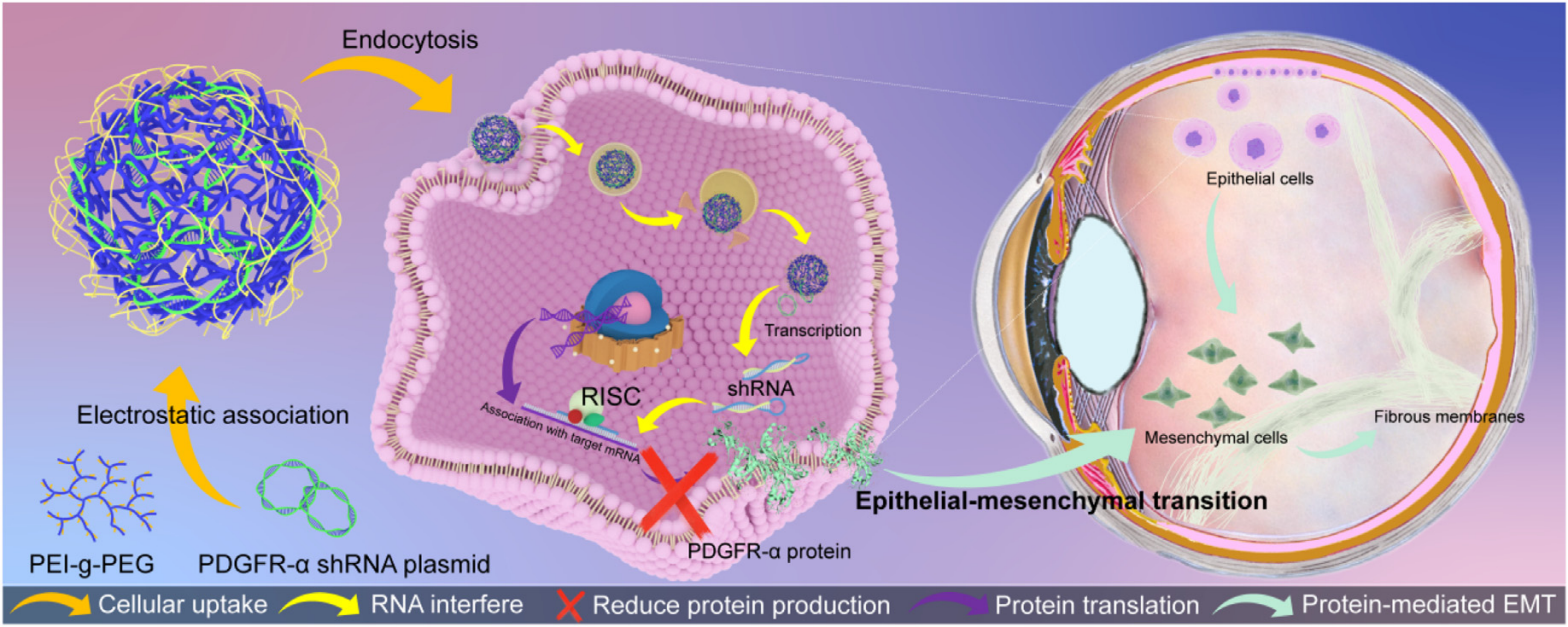
Schematic illustration based on polycationic PEI-*g*-PEG delivery of PDGFR-α shRNA plasmids for interfering with EMT to prevent vitreous cavity fibroproliferative membrane production.

Herein, PEI-g-PEG was selected as a non-viral nucleic acid therapeutic vector and PDGFR-α as a target interference site, to block the epithelial-mesenchymal transition process of cells. Furthermore, the establishment and treatment of PVR animal models showed that nanoscale non-viral vectors were ideal for RNAi and the silencing mode of PDGFR-α was effective in anti-fibrotic treatment and prevention of PVR (Scheme 1).

## 2 Materials and methods

### 2.1 Reagents and antibodies

PEI-g-PEG (PEI Mn ​= ​25,000, PEG Mn ​= ​2,000, the amount of PEG grafted is 2–3) was provided by Ruixi Bio. Polyethyleneimine (PEI Mn ​= ​25,000) and fluorescein isothiocyanate (FITC) were purchased from Sigma-Aldrich. Fetal bovine serum (FBS), Dulbecco's modified eagle's medium and Ham's F12 medium (DMEM/F12) cell culture media, 0.05% trypsin-EDTA, penicillin-streptomycin solution, and other cell culture-related reagents were purchased from Gibco. The cell counting kit-8(CCK-8), Hoechst 33,342, Calcein/PI Cell Viability/Cytotoxicity Assay Kit, 4% paraformaldehyde fix solution, 1,1′-dioctadecyl-3,3,3′,3′-tetramethylindocarbocyanine (DiI), Antifade Mounting Medium with DAPI, enhanced BCA protein assay (BCA) kit, Plasmid Maxi Preparation Kit for All Purpose and RIPA lysis buffer were provided by Beyotime Biotechnology Co. Phosphate-buffered saline (PBS) was purchased from Boster Biological Technology. A series of antibodies (Fibronectin, ZO-1, and PDGFR-α) used for western blotting (WB) was purchased from Santa Cruz and Immunoway. Fish sperm DNA was purchased from Aladdin. TGF-β1 was purchased from Sino Biological.

### 2.2 Cell culture and plasmid preparation

ARPE-19 ​cells were cultured with the complete medium at 37 ​°C in 5% CO_2_. The complete medium contained a 1:1 mixture of DMEM/F12 supplemented with 10% FBS and 1% penicillin-streptomycin solution. The medium was changed every 2–3 days. In other experiments, cells were trypsinized and seeded in 96-well or 24-well plates [9,55].

The reporter plasmid DNA, encoding an enhanced red fluorescent protein (RFP-pCAGGS) and green fluorescent protein (GFP-pCAGGS), was amplified in the lab and purified using the Plasmid Maxi Preparation Kit for all experiments [56]. The *Escherichia coli* clones containing recombinant plasmid were kindly provided by Prof. JG Chen (Wenzhou Medical University). The PDGFR-α shRNA plasmid DNA (PTSB–SH–mCherry-2A-NEO) was also propagated in *Escherichia coli* DH5α (Tsingke Biotechnology Co., Ltd., China). The concentration of DNA was determined by measuring the UV absorbance at 260 and 280 ​nm with the Ultraviolet–visible spectrophotometer (Denovix DS-11, America). The purified DNA was stored at −20 ​°C.

### 2.3 Fabrication and characterization of the gene complex

PEI-g-PEG/DNA (PP/DNA) complexes were obtained by electrostatic association. Briefly, PEI-g-PEG and DNA were dissolved in DEPC water (DNase/RNase free) at different N/P ratios (molar ratio of amino groups of PEI-g-PEG to phosphorus element of DNA). The solution was stirred for 30 ​s and left at room temperature for 30 ​min to form the PP/DNA complexes. PP/DNA complexes were characterized using fish sperm DNA as a DNA template [57].

The zeta potential and particle size of the gene complexes were analyzed by dynamic light scattering (Malvern Instrument Ltd, UK) at 25 ​°C ​at ​N/P of 1, 2, 5, 10, and 20, respectively. Moreover, the morphology of PP/DNA complexes (N/P ​= ​10) was analyzed by transmission electron microscopy (FEI Talos F200, America).

The ability of the copolymer to bind DNA was examined by gel electrophoresis assay. The PP/DNA complexes were prepared following the procedure described above. 10 ​μL suspensions of PP/DNA with different N/P ratios (1, 2, 5, 10, and 20) were run on a 2.5% (W/V) agarose gel containing 0.5 ​× ​TBE of NA-Red Buffer (160 ​V). The results were analyzed by using digital imaging systems (Bio-Rad GelDoc XR^+^, America) [58].

To test the stability of PP/DNA, the prepared solution was placed at 4 ​°C, and its particle size and potential were tested for 7 consecutive days. To simulate an in vitro serum stability test, PP/DNA solution was mixed with BSA, so that the final concentration of BSA in the solution was 10% (commonly used culture conditions for cell culture). The samples were continuously incubated for 7 days, and the same volume of each group was taken out for gel electrophoresis experiments.

### 2.4 In vitro cytotoxicity

The cytotoxicity of polycationic materials used for transfection was evaluated by a CCK-8 assay in ARPE-19 ​cells. The cells were incubated in a 96-well plate, at 5 ​× ​10^3^ ​cells per well with the complete medium. Then different polymer solutions of PEI-*g*-PEG were added to achieve the final concentrations of 2, 4, 6, 8, 10, 20, 30, 40, and 50 ​μg/mL. After 48 ​h of incubation, all the media in each well was replaced with 100 ​μL of fresh media. Subsequently, 10 ​μL of the CCK-8 reagent was added to each well, and the cells were incubated for 2 ​h at 37 ​°C. Absorbance was measured at 450 ​nm using a microplate reader (MD-SpectraMax M5, USA). The toxicity of polycationic materials with gradient concentration before and after PEG modification was also compared [59].

To further qualitatively analyze the toxicity of PEI-g-PEG and the nano complexes before and after PEG modification, cells were stained with Calcein/PI Cell Viability/Cytotoxicity Assay Kit after incubation for another 48 ​h [60]. Then cells were subjected to an inverted fluorescence microscope (Leica DMi8, Germany) and recorded fluorescence images. To compare the toxicity changes of polycationic materials as gene carriers before and after PEG modification, RPE cells were treated with gene complexes prepared under different N/P ratios (1, 2, 5, 10, 20) conditions. The nuclei were also stained with Hoechst 33,342 to observe changes in cell density to assess the magnitude of toxicity.

### 2.5 Cellular uptake of the gene complex

To assess cellular uptake, PEI-g-PEG was replaced by FITC-labeled PEI-g-PEG to prepare complexes. ARPE-19 ​cells were seeded in 24-well plates at a density of 4 ​× ​10^4^ ​cells/well and cultured for 24 ​h. After 4 ​h of starvation, replaced the original medium with a complete medium containing FITC-labeled PEI-g-PEG/DNA (P^FITC^P/DNA), and incubated for an additional 4 ​h [61]. For high-quality visualization of fluorescent images, cells were fixed with 4% paraformaldehyde for 30 ​min, stained with DiI and washed with PBS. The Antifade Mounting Medium with DAPI was added to each sample and the cells were sealed with cover glass [62]. Images of several fluorescence channels were acquired with a confocal laser scanning microscope (Zeiss LSM 880, Germany). Moreover, a quantitative calculation of cellular uptake efficiency was carried out. The main steps were cell filtration with 300 mesh, washing with PBS, and flow cytometry detection (BD FACSCalibur, America).

### 2.6 Establishment of cell EMT model

TGF-β was used to induce a cell model of EMT in ARPE-19 ​cells. Cells were seeded and cultured for 24 ​h and starved in a serum-free medium for 24 ​h. Then they were treated with gradient concentrations of TGF-β (0.5, 2.5, 10, 12.5 ​ng/mL) [63,64]. After 48 ​h of serum-free culture, the cell morphology was observed under a microscope. The CCK-8 kit was used to detect the promoting effects of different concentrations of TGF-β on cells.

### 2.7 The transfection of the gene complex

The PEI-g-PEG transfection complex with an N/P ratio of 10 as described above was prepared. Gene complexes loaded with different plasmid DNA encoding reporter genes were formed separately. They were named PEI-g-PEG/RFP (PP/RFP), PEI-g-PEG/GFP (PP/GFP), and PEI-g-PEG/PDGFR-α shRNA (PP/Sh). Among them, the PDGFR-α shRNA coding sequence is CCGGGCCAGCAATCTCTCAAATATTCTCGAGAATATTTGAGACATTGCTGGCTTTTTT.

The normal group and the epithelial-mesenchymal transition model group were set respectively. ARPE-19 ​cells were seeded in 24-well plates at the density of 4 ​× ​10^4^ ​cells/well. After 24 ​h of culture, the epithelial-mesenchymal transition model group was supplemented with TGF-β to a final concentration of 10 ​ng/mL. After 30 ​min of incubation at room temperature, the gene complexes (PP/RFP and PP/GFP) were added to 24-well plates and incubated for 48 ​h. The cells were then examined and images were taken under an inverted fluorescence microscope (Leica DMi8, Germany). To better monitor the cell microstructure after transfection, the samples of PP/GFP were selected with an N/P of 10 [65]. Subsequently, the cells were fixed with 4% paraformaldehyde for 30 ​min and stained lysosomes with Lyso-Tracker Red [66]. After washing with PBS, the Antifade Mounting Medium with DAPI was added to each sample, and the samples were sealed with cover glass. Images were recorded for several fluorescence channels with a confocal laser scanning microscope (Zeiss LSM 880, Germany).

In addition, to further explore the effect of PEI-g-PEG on gene transfection, flow cytometry was used to detect the effect of ARPE-19 ​cells on the red fluorescence expression in the normal cell group and the group of EMT.

### 2.8 Immunofluorescence (IF) analysis

ARPE-19 ​cells were seeded and cultured in a 24-well plate inlaid with cell slides. After the transfection of PP/Sh and TGF-β treatment, the cells were washed and fixed with 4% pre-cooling paraformaldehyde for 10 ​min. Then, cells were washed with PBS three times, permeabilized with 0.3% Triton X-100 and blocked with 5% BSA for 1.5 ​h at room temperature. Whereafter, cells were incubated with primary antibodies (Fibronectin and PDGFR-α) overnight at 4 ​°C. After washing three times with PBS, the samples were incubated with secondary antibodies for 1.5 ​h at room temperature [67,68]. Added the Antifade Mounting Medium with DAPI, and each sample was sealed with cover glass. Images were recorded for several fluorescence channels with a confocal laser scanning microscope (Zeiss LSM 880, Germany).

### 2.9 Western blot (WB) analysis

After treatment with TGF-β and PP/Sh, ARPE-19 ​cells were lysed in RIPA buffer on the ice for 30 ​min and quantified using the BCA protein assay kit. Fibronectin and PDGFR-α proteins were separated by polyvinyl fluoride membrane (PVDF) by polyacrylamide gel electrophoresis (SDS-PAGE), blocked with PBS-Tween 20 (PBST) nonfat dry milk, then stored at 37 ​°C for 1 ​h. Antibodies (PDGFR-α, FN, and GAPDH) were incubated overnight at 4 ​°C. The membrane was washed 5 times with PBST, incubated with the secondary antibody for 30 ​min at 4 ​°C and then washed once with PBST. Immunoreactive proteins were visualized on a gel imaging system (GE AI680, American) [69].

### 2.10 Establishment of the PVR model and treatment

The animal experiments were carried out following the approved experimental protocol and the regulations of the Laboratory Animal Ethics Committee of Wenzhou Medical University. The weight and intraocular pressure of the rabbits were routinely checked before the operation, and the rabbits with congenital eye diseases and mental disorders were excluded. Only one eye in each animal was used in the experiment. In each group (randomly assigned by random number table), 15 rabbits from 5 independent experiments (3 rabbits in each independent experiment) were included. PVR model group (called ARPE): ARPE-19 (1 ​× ​10^6^/mL 0.2 ​mL) cell suspension was injected into the vitreous cavity [70]. After injecting the cells, PP/Sh was injected from another location in the same eye. As the vitreous volume of rabbits was 1.2–1.5 ​mL [71], according to cytotoxicity and cell transfection experiments, the amount of plasmid transfected in the treatment group (called ARPE ​+ ​PP/Sh) was 4 ​μg. In the control group (labeled as PBS), the negative control group (labeled as ARPE ​+ ​PP/DNA), and the security testing group (labeled as PP/DNA), the injected DNA (or PEI-g-PEG) concentration and PBS volume remained unchanged to maintain the total amount of transfected DNA constant among the groups [72]. After 1, 3, 7, 14, 21 and 28 days, the fundus were examined with a digital fundus camera (Topcon TRC-50DX, Japan) and Spectral Domain OCT (Heidelberg Engineering, Germany) [9,73]. To study the biocompatibility in vivo, the ophthalmotonometer (Tono-Pen AVIA, USA) was used to measure the intraocular pressure of rabbits before the operation and 28 days after the operation [74]. Each eye was measured 3 times, and the reliability of each result was maintained above 95%. Electroretinogram (ERG) was used to record the electrophysiological function of the retina for comparison before and after treatment and between treated and normal eyes [75].

Subsequently, the rabbits were humanely sacrificed after 28 days. The eyeballs were removed and fixed immediately. Due to the size of the rabbit eye, the rabbit eye was partially incised along the sagittal plane and embedded in OCT glue. The tissue slice (10 ​μm) was prepared for hematoxylin-eosin staining (HE) and immunofluorescence staining for ZO-1 to assess the degree of retinal detachment [9]. Immunofluorescence staining for Fibronectin and PDGFR-α of ocular tissues was also performed using the same method to assess EMT. Other tissues of the eye (cornea, iris) were evaluated by histological sections and HE staining to evaluate the safety of the treatment procedure [76].

### 2.11 Statistical analysis

Three to five duplications for each sample were set in the experiment. The results were expressed as mean ​± ​standard error of the mean. Each experiment was repeated three times independently. Statistical analysis and one-way analysis of variance were performed using GraphPad Prism 7 (San Diego, California). The significance level was set at p ​< ​0.05. Differences with p ​< ​0.05 (∗) were considered to be significant, those with p ​< ​0.01 (∗∗) and p ​< ​0.001 (∗∗∗) were considered to be very significant and those with p ​> ​0.05 were considered to be nonsignificant.

## 3 Results and discussion

### 3.1 Characterization of the gene complex

**Fig. 1:**
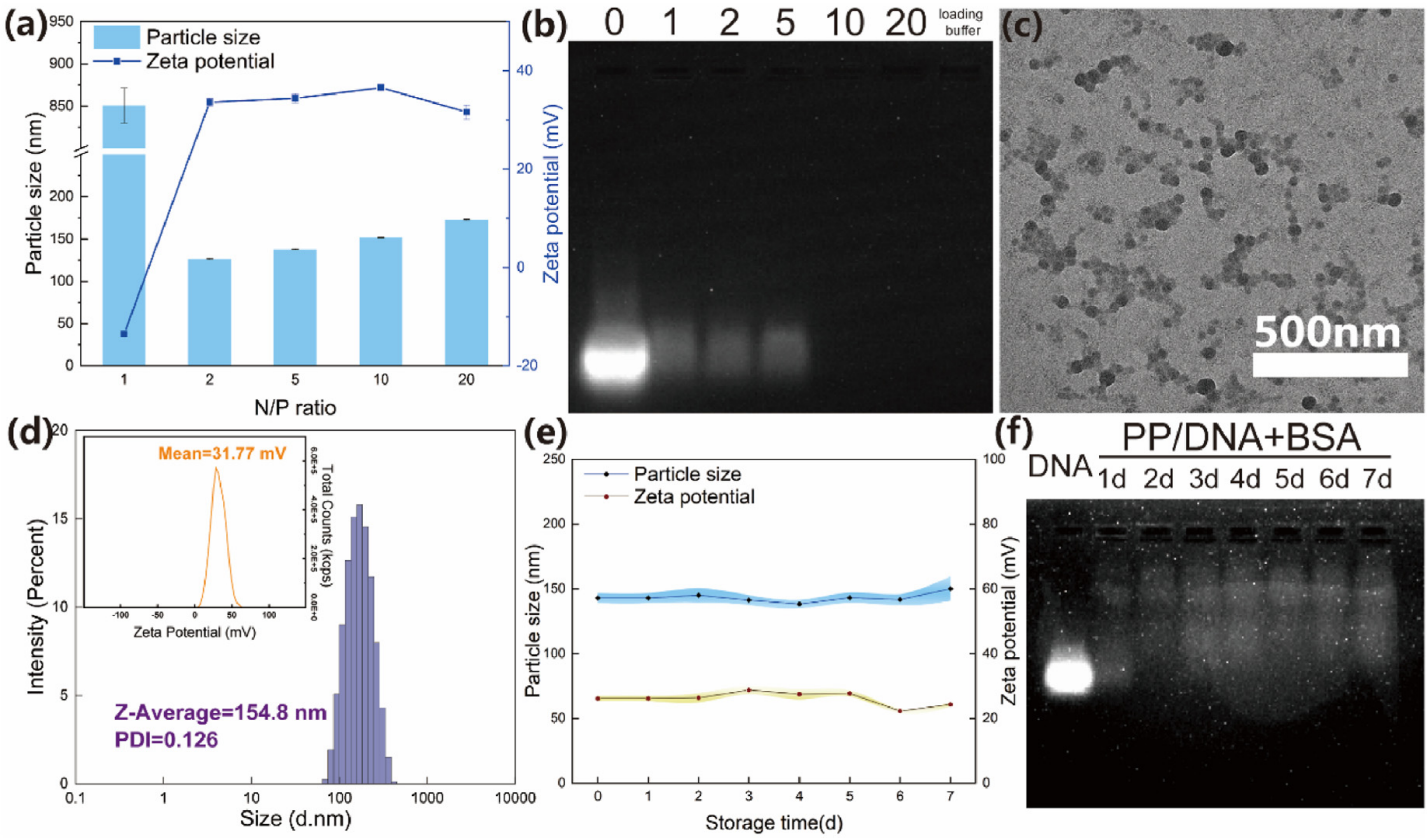
Particle size, zeta potential (a), and agarose gel electrophoresis diagram (b) of the PP/DNA were obtained at different N/P ratios. TEM image (c) of PP/DNA obtained at N/P ​= ​10. (d) Particle size and Zeta potential of PP/DNA under the N/P ratio of 10. (e) Changes in particle size and Zeta potential of PP/DNA for 7 days. (f) Nucleic acid leakage from PP/DNA co-incubated with BSA for 7 days.

Particle size and zeta potential directly affect the stability, cellular uptake, biodistribution, and transfection efficiency of gene complexes [[77], [78], [79]]. As shown in Fig. 1, as the most widely used non-viral gene delivery carrier among cationic polymers, PEI on the backbone of polymer PEI-*g*-PEG can interact with the negatively charged DNA through electrostatic interactions action to form complexes [80,81]. Fig. 1a showed that the diameter of the complex increased with the increase of N/P but tended to about 150 ​nm and the zeta potential tended to +34 ​mV. Both provided a solid foundation for better binding of the transfection complex to negatively charged cell membranes in vivo. It is worth noting that when N/P was 1, the diameter was as high as 850 ​nm. Probably due to an insufficient supply of PEI-g-PEG, the DNA in the solution was still relatively dispersed, and coiled loosely around the positively charged core PEI. Meanwhile, the potential of negative charge also proved this hypothesis. When N/P ​> ​10, the particle size was increased, but the potential decreased slightly. This may be the loose distribution of particles owing to the lack of negatively charged nuclei [82]. Therefore, the selection of appropriate N/P is particularly important for the subsequent experimental process, so an agarose gel electrophoresis experiment was carried out. As shown in Fig. 1b, when the N/P was greater than or equal to 10, no obvious DNA bands were found, indicating that the DNA fragments had been completely encapsulated inside the particles by the positively charged PEI-g-PEG, making NA-Red unable to bind to the nucleic acid used for imaging. It also indicated that there would be surplus PEI-g-PEG in the solution when a ratio greater than 10. The band brightness of the group without PEI-g-PEG addition (N/P ​= ​0) was significantly higher than that of the subsequent groups, which also proved that polycation materials partially wrapped DNA. Therefore, PP/DNA with N/P greater than or equal to 10 has a higher DNA wrapping efficiency.

Fig. 1c showed the morphology of the electrostatic association from the microstructure, in which the PP/DNA complex presented a spherical shape with good dispersion. Meanwhile, Fig. 1d showed that the size distribution of PP/DNA (N/P ratio ​= ​10), the hydrated particle size was 154.8 ​nm, and the polydispersity index (PDI) was 0.126. This means that the gene complex preparation has good dispersibility [83]. The Zeta potential of PP/DNA was +31.77 ​mV, which further facilitated the interaction with the negatively charged cell membrane, and enhances cellular uptake [84].

From Fig. 1e, it could be seen that the particle size of the PP/DNA (N/P ​= ​10) fluctuates around 150 ​nm, and its potential was still positive for 7 days. Accordingly, PP/DNA in an aqueous solution had good storage stability at 4 ​°C. The gel electrophoresis results in Fig. 1f showed that no significant leakage of nucleic acid inside the PP/DNA was observed in consecutive 7 days, indicating that it also had great serum stability for the delivery of gene fragments.

### 3.2 In-vitro cytotoxicity

**Fig. 2:**
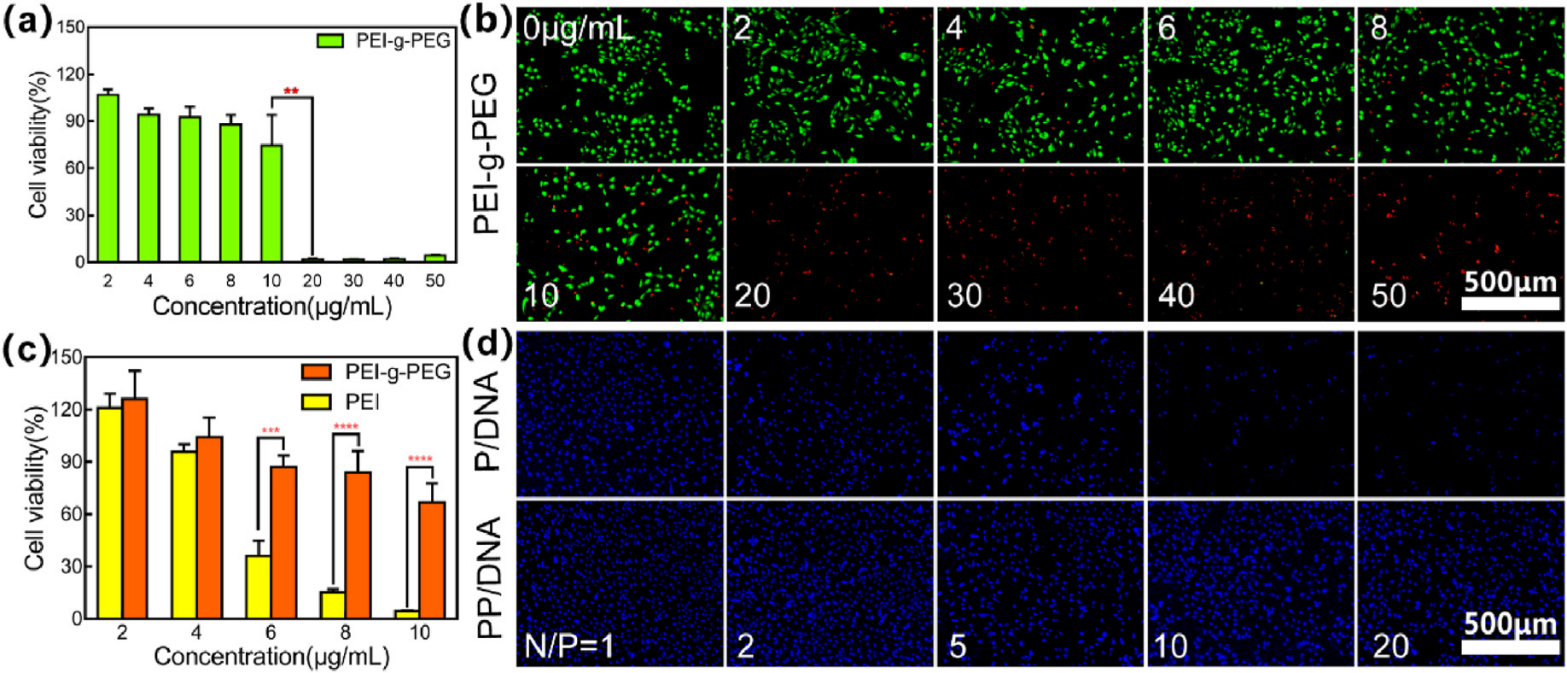
Cell viability (a) and representative Calcein/PI stained cell images (b) of PEI-g-PEG at different concentrations. (c) Comparison of the toxicity of PEG-g-PEI and PEI at different concentrations. ∗p ​< ​0.05, ∗∗p ​< ​0.01, ∗∗∗p ​< ​0.001. (d) Comparison of the toxicity of gene complexes formed by two materials before and after PEG modification under different N/P.

The primary evaluation condition of polycation materials as transfection reagents is whether they are safe and non-toxic to cells [19]. Therefore, ARPE-19 ​cells were co-incubated with PEI-g-PEG at different concentrations for 48 ​h to observe the toxicity of the selected non-viral vectors on the cells. Qualitative and quantitative analysis were conducted for each group of cells after co-culture successively. In Fig. 2a, it was shown that the cell viability of PEI-g-PEG treated with different concentrations also showed a gradient decline, especially when the concentration was above 10 ​μg/mL, the cell viability showed a cliff decline. It can be seen that PEI-g-PEG concentration should be kept below 10 ​μg/mL, and cell viability could be kept above 80% if it was kept below 8 ​μg/mL. The results shown in Fig. 2b were consistent with the above-mentioned qualitative analysis conclusions. When the concentration was 10–50 ​μg/mL, more red fluorescence (PI staining, dead cells) appeared and the corresponding green (Calcein AM staining, living cells) was sparse and fragmented, indicating that the cell state was very poor.

The introduction of the PEG group can reduce the cytotoxicity of PEI [34]. Therefore, the same treatment was used to detect the cytotoxicity of the material before and after PEG modification. As shown in Fig. 2c, the cell viability of PEI in all groups was lower than PEI-g-PEG and it was even lower than 50% at 6 ​μg/mL, indicating that PEI-g-PEG is more suitable for transfection studies. This is mainly because since the cationic charge of the polymer is shielded, PEGylation can significantly reduce the toxicity of PEI [85,86].

In addition, we also tested the influence of the two materials on cells after electrostatic interactions at different N/P (1, 2, 5, 10, 20). It could be seen that the cell density of both P/DNA and PP/DNA decreased with the increase of N/P, which meant that after the formation of complexes, the cytotoxicity mainly depends on the content of polycation material. This also explained the reason that the number of cells in the P/DNA group was less than PP/DNA under the same N/P condition. In contrast, there was little difference in cell density between PP/DNA groups, indicating that PP/DNA was more suitable for cell transfection.

### 3.3 Cellular uptake of gene complex in vitro

**Fig. 3:**
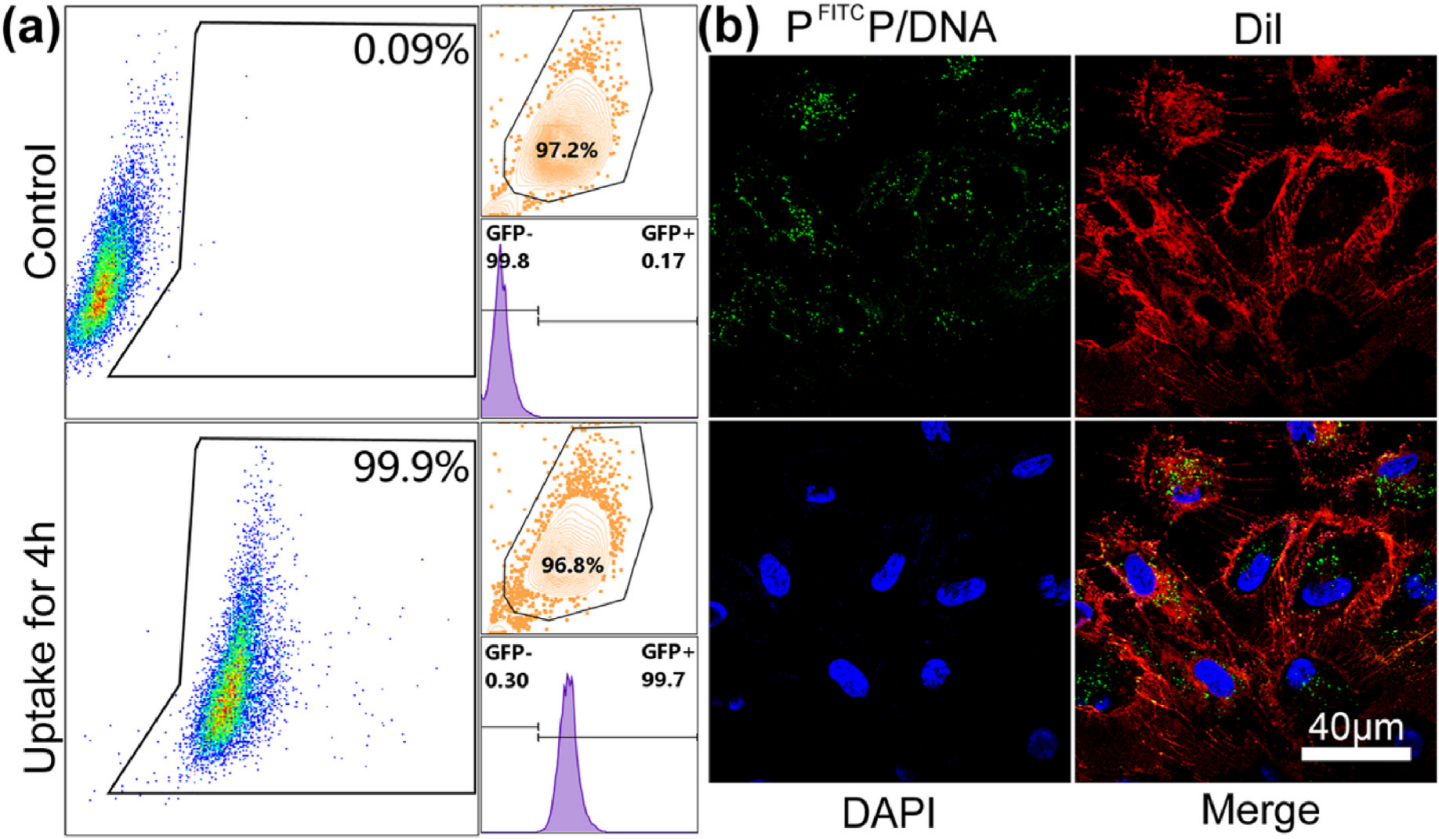
(a) Scatter plots and histogram recorded by flow cytometry of P^FITC^P/DNA treated ARPE-19 ​cells. (b) Confocal laser microscopic images of ARPE-19 ​cells with P^FITC^PD incubation.

The efficiency of cellular uptake directly determines the efficacy of gene and drug delivery [[87], [88], [89]]. To facilitate the study of the uptake ability of ARPE-19 ​cells to transfected particles, we carried out FITC labeling on PEI-g-PEG, and the formed complexes were named P^FITC^P/DNA (N/P ​= ​10). Fig. 3a showed the flow detection results of ARPE-19 ​cell uptake. Compared with the control group, the uptake effect of gene complexes could be as high as 99% after 4 ​h of transfection (99.9%–0.09% in the scatter plot, 99.7%–0.17% in the gray value map). All of these demonstrated the excellent uptake ability of the formed P^FITC^P/DNA complexes by the cells, which was mainly due to the suitable particle size of the gene complex and the electrostatic attraction of the positively charged shell to the negatively charged cell membrane surface. To observe the cellular uptake more intuitively, the microstructure of cells incubated with ARPE-19 and P^FITC^P/DNA for 4 ​h was observed under a laser confocal microscope. As shown in Fig. 3b, P^FITC^P/DNA appeared as green granules, DiI stained the ARPE-19 ​cell membrane, and the structure of the cell membrane and the protruding pseudopodia shape could be observed, the nucleus was stained to blue. The merged channel showed that the green complexes fill the interior of the cytoplasm and surround the nucleus. The above results show that P^FITC^P/DNA can be endocytosed by cells quickly and efficiently, which is beneficial to the next step of gene transfection and treatment of diseased cells.

### 3.4 The establishment of the EMT model

**Fig. 4:**
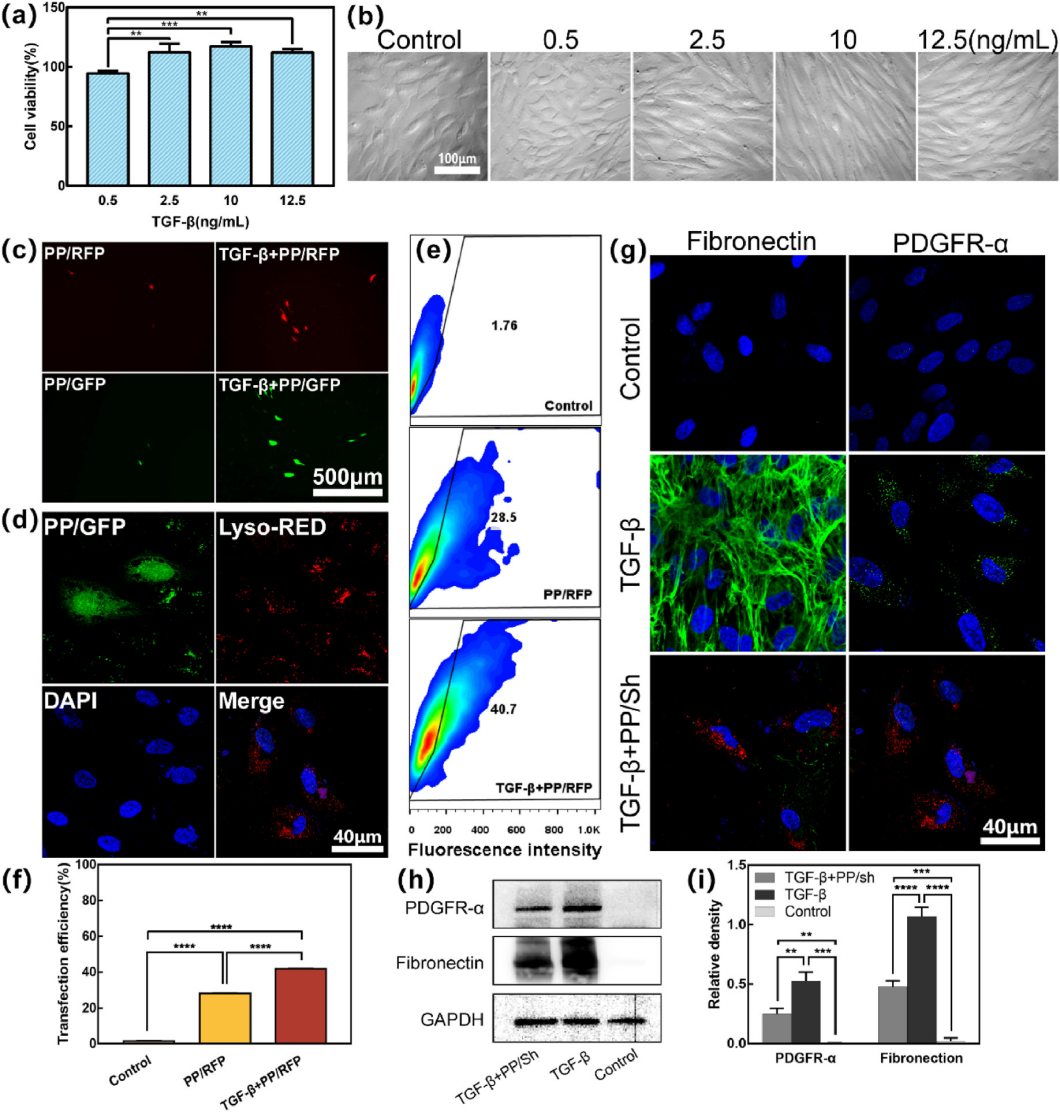
(a) Cell viability of ARPE-19 treated with different concentrations of TGF-β (0.5, 2.5, 10 and 12.5 ​ng/mL). (b) Cell morphology of ARPE-19 treated with different concentrations of TGF-β (c) Transfection effect of PEI-g-PEG gene vector (N/P ​= ​10) with GFP (PP/GFP) and RFP (PP/RFP) on ARPE-19 ​cells. (d) Confocal laser scanning microscopy images of ARPE-19 ​cells incubated with PP/GFP. (e) Flow cytometry was used to record the scatter diagram of ARPE-19 ​cells transfected with PP/RFP and TGF-β+PP/RFP. (f) Cell transfection efficiency quantification of (e). (g) Immunofluorescence analysis of EMT-related protein (fibronectin) and targeted silencing protein (PDGFR-α) in recipient ARPE-19 ​cells. Nuclei were stained with DAPI. (h) The protein expression of PDGFR-α and fibronectin was detected by Western blot. (i) Quantification of Fig. 4h.

We used different concentrations of TGF-β (0.5, 2.5, 10, and 12.5 ​ng/mL) to induce epithelial-mesenchymal transformation of ARPE-19 ​cells. Fig. 4a showed the effect of different concentrations of TGF-β on cell proliferation. The cell viability of groups with concentrations of 2.5, 10 and 12.5 ​ng/mL were observed to be greater than 100%, indicated that these concentrations had the effect of stimulating cell proliferation. There were significant differences between the 0.5 ​ng/mL group and the group of 10 ​ng/mL had the most obvious effect on promoting cell proliferation. Similarly, we conducted a morphological observation of cells in each group under the bright field (Fig. 4b). It could be seen that, with the increase of TGF-β, ARPE-19 ​cells were arranged gradually closer, and the shape changed into a spindle needle shape, which was the most obvious in the 10 ​ng/mL group. After comprehensive consideration, 10 ​ng/mL was selected as the optimal concentration for EMT model establishment in subsequent experiments [7,9,55].

### 3.5 Transfection effect study

To investigate the effect of PEI-g-PEG on gene delivery and further transfection, we replaced fish sperm DNA with the plasmids encoded green fluorescent protein fragments and red fluorescent protein and named PP/GFP and PP/RFP respectively [65]. Fig. 4c showed that PEI-g-PEG could provide a better transfection effect for both model plasmids and cells in the epithelial-mesenchymal transition model group are easier to express corresponding functional fragments. Fig. 4d showed the microscopic situation after PP/GFP (N/P ​= ​10) transfection. Green signal represented the expression of green fluorescent protein after transfection. The lysosomal was stained to red of each cell for convenient localization and the nucleus was shown in blue. It could be found that there were clumps and granulations in the fluorescence expression, which might be due to the different expression amount and time of the coding green fluorescent fragment in each cell.

Fig. 4e and f showed the red fluorescent protein expression in ARPE-19 ​cells in normal (PP/RFP) versus EMT state (TGF-β+PP/RFP), and the PEI-g-PEG transfection effect was quantified using flow cytometry counts. The statistics of the number of positive cells in Fig. 4f showed that the cells in PP/RFP group and TGF-β+PP/RFP group had different degrees of red fluorescence expression, and the transfection effect of cells in the EMT state was better, 41.03 ​± ​0.29%, which was much higher than that of PP/RFP group (29.07 ​± ​0.25%). The statistic confirmed that ARPE-19 ​cells in the EMT state are more sensitive to PP/RFP transfection, providing a strong opportunity for subsequent delivery of therapeutic gene fragments.

### 3.6 Gene silencing effect study

In addition, immunofluorescence analysis was performed for fibronectin and silenced target proteins in EMT markers in the TGF-β treatment group (TGF-β) and PDGFR-α interference group (TGF-β+PP/Sh) [9,55]. As seen in Fig. 4g, no related proteins were expressed in the control group (green signal), whereas the fibronectin was highly expressed in the TGF-β treated group and PDGFR-α was punctate expression. After gene silencing, the expression of fibronectin and PDGFR-α were down-regulated to varying degrees, and each cell expressed red fluorescence of mCherry, which was encoded on the same plasmid as the silenced fragment. To quantify the silencing effect, Western blot analysis was also performed on the protein level (Fig. 4h). There were significant differences when comparing the three groups (Fig. 4i), indicating that PP/Sh downregulates the expression of PDGFR-α and fibronectin in EMT cells. In conclusion, down-regulation of PDGFR-α expression by PP/Sh can affect the epithelial-mesenchymal transformation of ARPE-19 at the cellular level.

### 3.7 Establishment of the PVR model and treatment

**Fig. 5:**
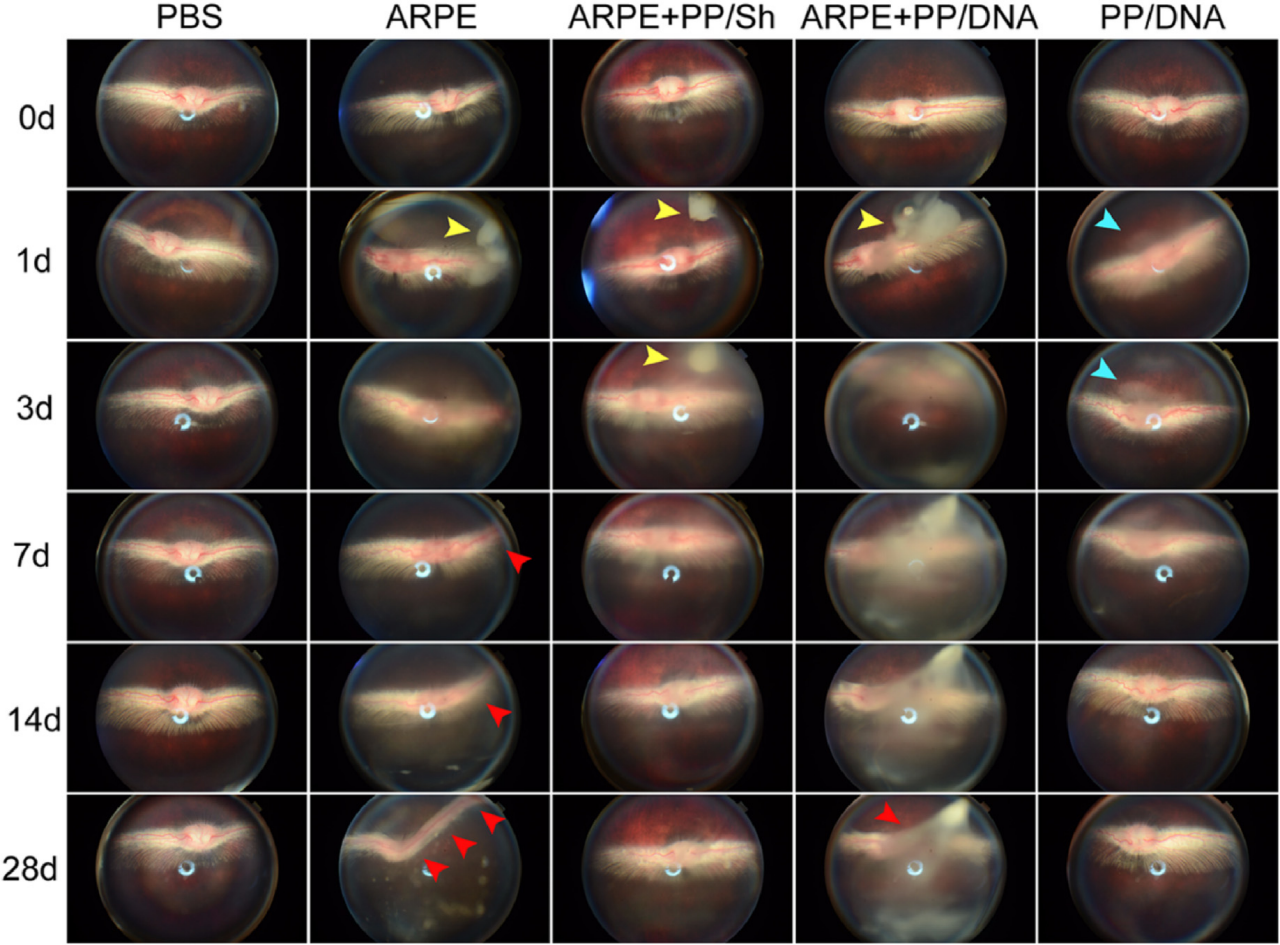
Representative images of the fundus. From left to right, control group: vitreous cavity injection of PBS (named PBS), PVR modeling group: injection of ARPE-19 (called ARPE), treatment group: injection of ARPE-19 with PEI-g-PEG/PDGFR-α shRNA (named ARPE ​+ ​PP/Sh), negative control group: injection of ARPE-19 with PEI-g PEG/fish sperm DNA (called ARPE ​+ ​PP/DNA), biosafety group: injection of PEI-g-PEG/fish sperm DNA only (called PP/DNA). Yellow arrows represent ARPE-19 ​cell clusters, red arrows represent retinal retraction and fibrous proliferation membranes, and blue arrows represent PP/DNA fluid. (For interpretation of the references to color in this figure legend, the reader is referred to the Web version of this article.)

Fig. 5 showed the characteristic fundus images of the five groups followed continuously for 28 days [70]. The visual field of the control group (PBS) was clear and translucent, the optic disc and blood vessels were visible, and the retina showed a homogeneous dark red color. In contrast, the PVR model group (ARPE), the treatment group (ARPE ​+ ​PP/Sh) and the negative control group (ARPE ​+ ​PP/DNA) all had visible cell clumps on the first postoperative day (indicated by yellow arrows). The vitreous cavity became cloudy on the third postoperative day, but the diffuse behavior of the cell clumps was significantly lighter in the treatment group than in the other two groups. The PVR model group was observed at 7, 14, and 28 days. In the PVR model group, the blood vessels and retina around the optic disc were gradually stretched (red arrows) and eventually formed a “tick” shaped optic disc. In contrast, in the treatment group (ARPE ​+ ​PP/Sh), the mild vitreous clouding that existed in the first three days gradually dissipated with metabolism, and the visual field became clearer. In the negative control group, however, the turbidity became more severe. Although the peripheral visual field became somewhat clearer, the epiretinal membrane (red arrows) gradually formed, affecting the deeper observation of the fundus, which needed to be observed in conjunction with subsequent OCT images. The last group (PP/DNA) of the vitreous cavity was injected with only the gene complex to perform in vivo toxicity studies. A cloudy appearance of fluid in the visual field (blue arrow) could be seen on the first day and the third day, and gradually becomes clearer in subsequent observations, which should be caused by the presence of PP/DNA fluid. By and large, it appeared that the gene particles do not affect the rabbit eyes.

**Fig. 6:**
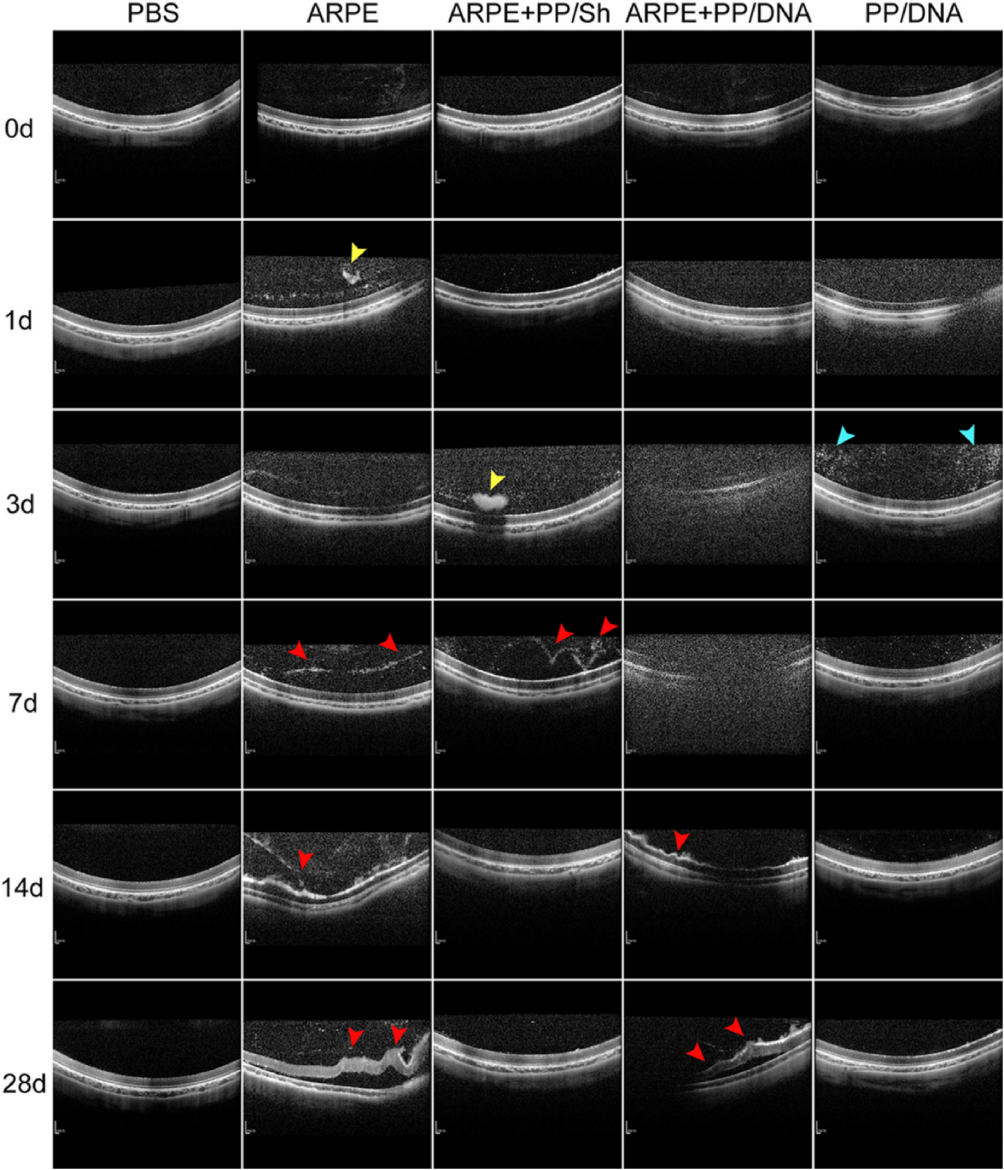
Representative OCT images of the eyes. From left to right, control group: vitreous cavity injection of PBS (named PBS), PVR modeling group: injection of ARPE-19 (called ARPE), treatment group: injection of ARPE-19 with PEI-g-PEG/PDGFR-α shRNA (named ARPE ​+ ​PP/Sh), negative control group: injection of ARPE-19 with PEI-g PEG/fish sperm DNA (called ARPE ​+ ​PP/DNA), biosafety group: injection of PEI-g-PEG/fish sperm DNA only (called PP/DNA). Yellow arrows represent ARPE-19 ​cell clusters, red arrows represent retinal retraction and fibrous proliferation membranes, and blue arrows represent PP/DNA fluid. (For interpretation of the references to color in this figure legend, the reader is referred to the Web version of this article.)

In addition to the extensive observation of the fundus, an OCT plain scan of the cross-section of rabbit eyes was also performed (Fig. 6). 10 layers of the retina were visible and the vitreous cavity was translucent in the PBS group. While the PVR model group (ARPE) and the treatment group (ARPE ​+ ​PP/Sh) showed clumpy shadows (yellow arrows) on day 1 and day 3, respectively. In the model group, there were different degrees of fibrous retractions on days 7–28, and the retina showed slight folds on day 14, while the retinal detachment was evident on day 28 (red arrows). In the treatment group (ARPE ​+ ​PP/Sh), except for the localized fibrous membrane on day 7 (red arrow), clear visual fields and intact retinas were observed on days 14 and 28. In the control group (ARPE ​+ ​PP/DNA), the imaging quality decreased to varying degrees from day 3. On days 14 and 28, due to the occlusion of the intravitreal membrane (corresponding to fundus photography), only half of the retinal morphology could be photographed, and retinal detachment similar to that of the model group could be seen. The in vivo toxicity group (PP/DNA) underwent a process of slight granular clouding to clarity. Overall OCT results corresponded to fundus photography, and PP/Sh showed good treatment effects.

### 3.8 HE staining and tissue immunofluorescence analysis

**Fig. 7:**
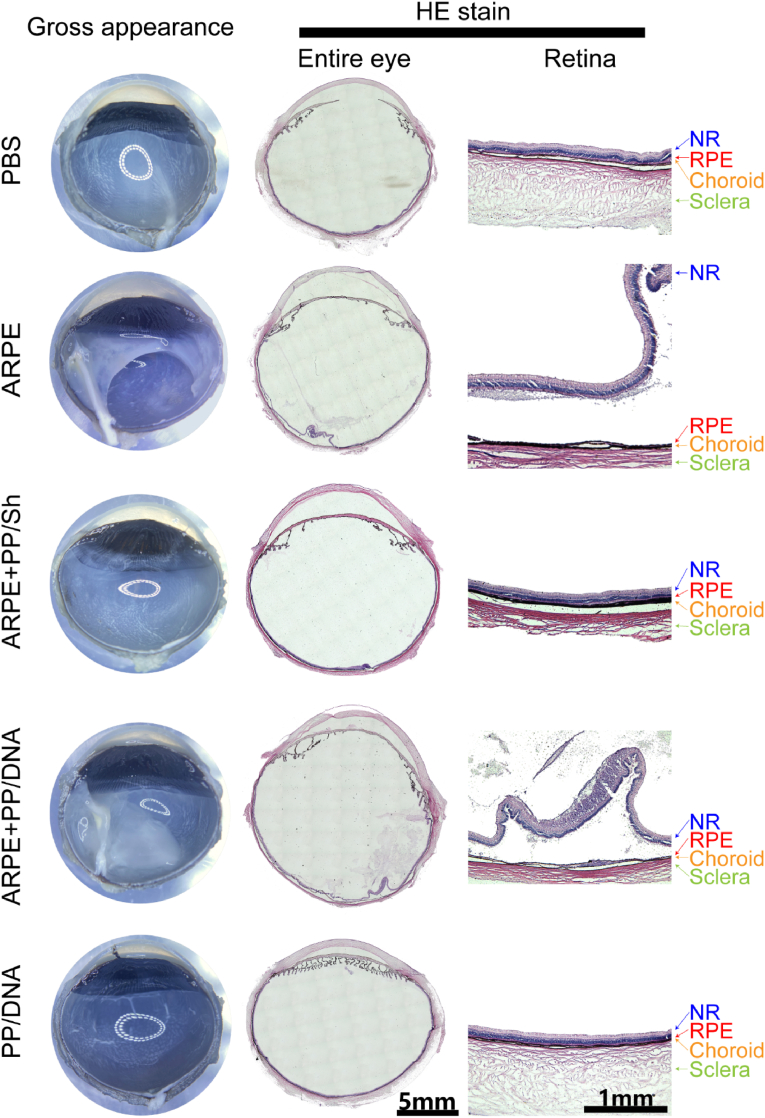
Representative images of the gross appearance and histological analysis. From top to bottom, the experiment is grouped as before. From left to right, the naked eye view of the sagittal section, HE staining of the whole eye, and retina, respectively. NR: Neurosensory retina, RPE: Retinal pigment epithelium

The whole eye was observed grossly (Fig. 7) and it was seen that both the modeling group (ARPE) and the negative control group (ARPE ​+ ​PP/DNA) formed intravitreal membranous material articulating with the serrated rim and retina. It was seen that the modeling group even formed a white bundle from the optic disc to the serrated rim. Later, the tissues were frozen and analyzed by HE staining, and it could be seen that the inside of the vitreous cavity of the three groups of PBS, ARPE ​+ ​PP/Sh, and PP/DNA in the whole-eye view was relatively clean. The retina was relatively tight in all parts and the retinal local pictures showed that the ten layers of the retina were tightly articulated without retinal detachment, and the morphology of each tissue was normal. While in the whole-eye view of the ARPE and ARPE ​+ ​PP/DNA groups, fibrous membranes and cellular masses were faintly visible in the posterior pole. Local magnification of the retina showed detachment of the anterior 9 layers of the retina (collectively referred to as the retinal sensory layer) from the retinal pigment epithelium (purple-black layer), and severe folds were formed. HE staining of the tissues showed that the use of PEI-g-PEG as a transfection vector was effective in treating the formation of PVR in vivo.

**Fig. 8:**
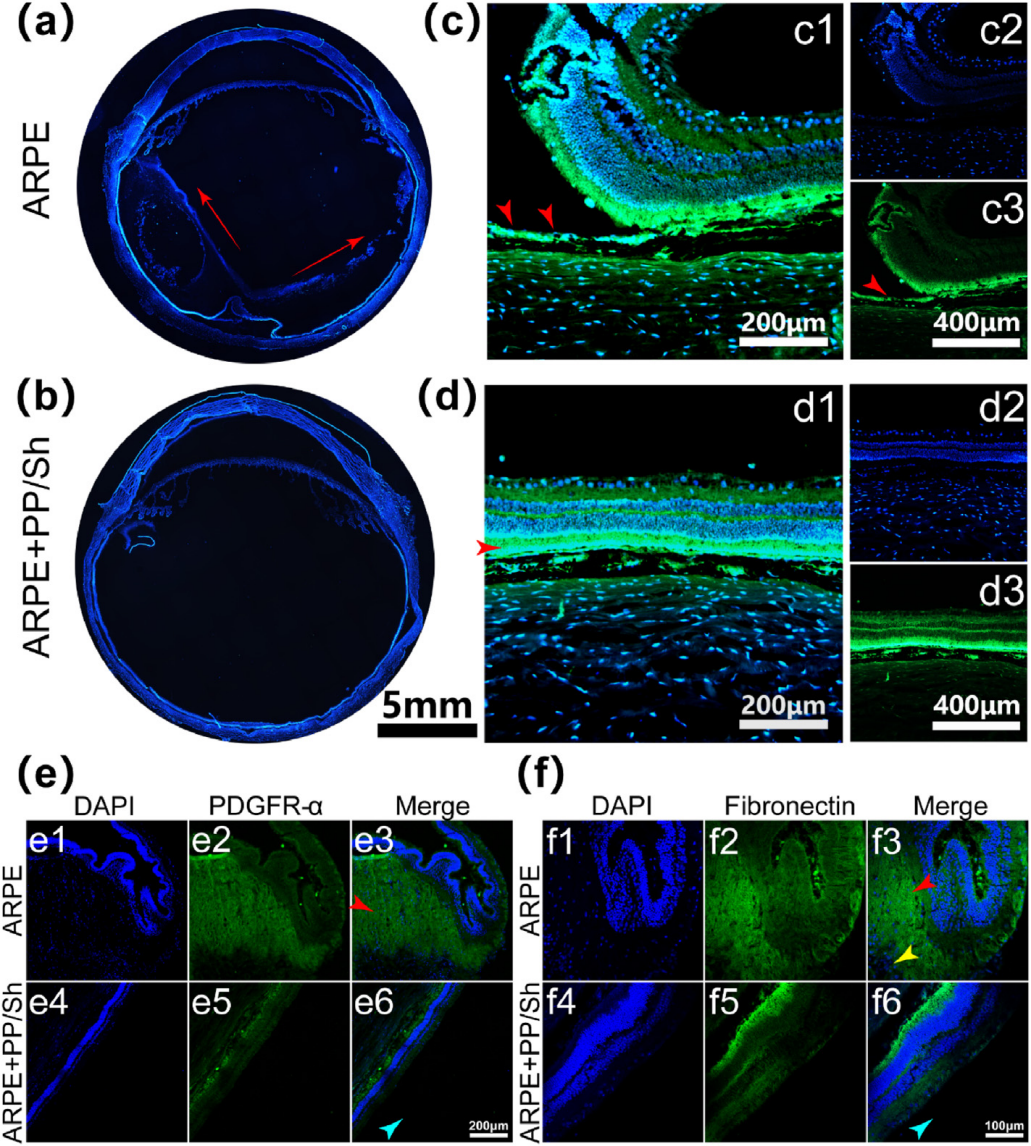
Representative images of the immunofluorescence assays. (a–d) ZO-1 was used to indicate the tight junctions of the cells of the retinal layers between the PVR model group and the treated rabbits, thus highlighting the retinal detachment shape. The nuclei are blue with DAPI staining (a, b), the red arrow marks the fibrous membrane of the vitreous cavity (a), and the red arrow represents the retinal pigment cell layer (c, d). (e, f) The expression of PDGFR-α and Fibronectin in the ARPE group and ARPE ​+ ​PP/Sh group were shown, respectively. (For interpretation of the references to color in this figure legend, the reader is referred to the Web version of this article.)

Fig. 8 showed the immunofluorescence results of the characteristic tissue sections of the PVR model and treatment groups. Fig. 8a showed the whole-eye stained image, where the galaxy-like fibrous proliferating membrane could be seen in the model group converging from the posterior pole toward the serrated edge on both sides (red arrows) and pulling the posterior pole to form folds and produce detachment. In Fig. 8c, for localized observation, the tight junctions between cells were stained using zonula occluden-1 (ZO-1) (c3 green) The most obvious fluorescence of ZO-1 between the retinal pigment epithelium and photoreceptor cells can be seen, while DAPI stains the nuclei between the outer nuclear layer, inner nuclear layer and ganglion cell layer (c2 blue). In the part of the c1 overlap channel, there was a layer of green fluorescence (red arrow) closest to the choroid. In combination with the HE-stained picture, it was the retinal pigment epithelium, which more visually showed the retinal detachment. In contrast, the vitreous cavity in the treated group in (b) appears particularly clean. While local observation (d1) showed tight connections between the layers, and the retinal pigment epithelium (red arrow) is visible and without folds. In (d3), in addition to the retinal pigment epithelium and columnar and cone photoreceptors, the tight connections between the cells of the inner and outer plexiform layers and the nerve fiber layer were also more obvious than in (c3).

In Fig. 8 e3, the green fluorescence in the fibrous membrane was significantly increased in the ARPE group (red arrows), while no green fluorescence was expressed in the vitreous cavity in ARPE ​+ ​PP/Sh (blue arrows). This result demonstrated the overexpression of PDGFR-α in the fibrous membranes of PVR and also confirmed the successful interference with PDGFR-α protein by the PP/Sh gene complex. For the EMT marker, in the ARPE group (Fig. 8 f3), a large area of green fluorescence representing Fibronectin (red arrows) could be seen near the retinal detachment site, while the presence of blue nuclei (yellow arrows) could be seen in the inner vitreous cavity away from the retina and the green fluorescence is significantly diminished. In the RPE ​+ ​PP/Sh group (Fig. 8 f6), the vitreous cavity was cleaner and free of residual cells, while there was no expression of green fluorescence. This result suggested that the EMT-RPE does overexpress Fibronectin to form a fibrous membrane to pull the retina and that the expected anti-fibrotic effect of PP/Sh is also present.

### 3.9 In vivo biocompatibility evaluation

**Fig. 9:**
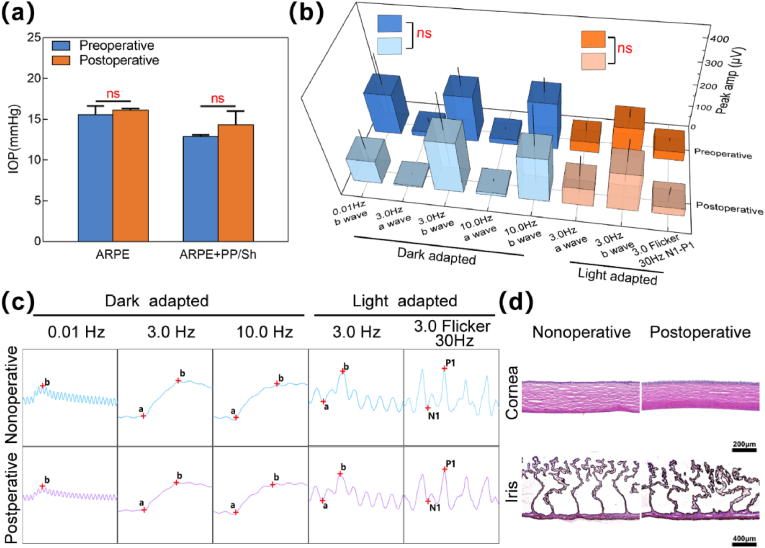
In vivo biocompatibility evaluation. (a) The intraocular pressure examination of eyes in the ARPE group and ARPE ​+ ​PP/Sh group. (b) Statistical analysis of the amplitudes of waves at different stages of ERG before and after treatment in ARPE ​+ ​PP/Sh. (c) ERG of nonoperative (light eye, blue) and operative eyes (right eye, purple) in the ARPE ​+ ​PP/Sh group. (d) Representative images of HE-stained tissue involving cornea and iris. (For interpretation of the references to color in this figure legend, the reader is referred to the Web version of this article.)

Intraocular pressure (IOP) is the pressure exerted by the contents of the eyeball on the wall of the eye. Under normal circumstances, the intraocular pressure is stable within a certain range to maintain the normal shape of the eyeball and maintain a good refractive state at the interface of each refractive medium [90]. The statistical analysis of IOP is shown in Fig. 9 (a). The intraocular pressure of each group was within the normal range before and after the operation, and there was no significant difference, indicating that the establishment of the animal model and treatment procedure did not affect the intraocular pressure.

The electroretinogram (ERG) is a complex electrical response that indicates retinal function [91]. As shown in Fig. 9 (b), the ERG of operative eyes in the ARPE ​+ ​PP/Sh group were analyzed before and after treatment. Statistical analysis was performed on the amplitudes of every wave in both dark and light adaptation, and there was no significant statistical difference at any stage. The results showed that retinal function did not change significantly after the treatment procedure of PP/Sh and there was no signs of retinal detachment. What's more, the ERG of normal eyes (left eye) and operative eyes (right eye) in the ARPE ​+ ​PP/Sh group were analyzed in Fig. 9 (c). The ERG curves of the left and right eyes were the same in both dark and light adaptation, and there was no significant difference in the occurrence time and amplitude of the peak (b wave) and trough (a wave). This suggested that retinal function after PP/Sh treatment was indistinguishable from healthy eyes.

The representative histological section images of the cornea and iris in Fig. 9 (d) demonstrated that PP/Sh treatment did not cause histomorphological damage to surrounding tissues. All of these results proved that the procedure of PP/Sh exhibits good biocompatibility and biosafety.

## 4 Conclusion

In this study, the polycationic PEI-*g*-PEG was selected as a non-viral gene carrier to deliver nucleic acid molecules to cells. The N/P ratio of the gene complex was a key factor affecting the particle size, surface electrical properties and the performance of electrostatic association. When N/P was greater than or equal to 10, it could effectively wrap DNA into cationic complexes with high cellular endocytosis properties. After the graft of polyethylene glycol, it could be used as a transfection reagent with simple fabrication, reproducibility and good biocompatibility.

In addition, the PDGFR-α shRNA plasmid was delivered into ARPE-19 ​cells. It inhibited the expression of corresponding target proteins and significantly affected their epithelial-mesenchymal transition process by RNAi technology. In a proliferative vitreoretinopathy model, this method was also effective against the fibrous membrane formation in vivo.

Thus, the successful use of advanced nanotechnology for nucleic acid therapy offers a possibility for future clinical use in non-viral vectors and also confirms that the prevention of proliferative vitreoretinopathy can be achieved by affecting the epithelial-mesenchymal transition process.

## Credit author statement

Jiahao Wang: Investigation, Data curation, Methodology, Formal analysis, Writing - original draft. Peiyi Zhao: Investigation, Validation. Zhirong Chen: Investigation. Hui Wang: Investigation. Yajia Wang: Investigation. Quankui Lin: Conceptualization, Funding acquisition, Project administration, Resources, Supervision, Writing-review & editing.

## Declaration of competing interest

The authors declare that they have no known competing financial interests or personal relationships that could have appeared to influence the work reported in this paper.

## Data Availability

Data will be made available on request.
